# Risk assessment of Cd exposure through consumption of jasmine rice

**DOI:** 10.1016/j.toxrep.2026.102242

**Published:** 2026-03-23

**Authors:** Supalak Kongsri, Chunyapuk Kukusamude

**Affiliations:** Nuclear Technology Research and Development Center (NTRDC), Thailand Institute of Nuclear Technology (Public Organization), 9/9 Moo 7, Saimoon, Ongkharak, Nakhon Nayok 26120, Thailand

**Keywords:** Cadmium, Estimated monthly intake, ICP-MS, Non-carcinogenic risks, Simulation

## Abstract

Concentrations of cadmium (Cd) in 46 jasmine rice samples were measured by inductively coupled plasma mass spectrometer (ICP-MS). The rice samples were digested using microwave-assisted acid digestion prior to ICP-MS analysis. The recovery for the analysis of Cd in rice samples was higher than 80%. The mean Cd concentration found in jasmine rice samples of 0.0119 mg kg^−1^ was obtained. Cd concentration in the studied samples was lower than 0.4 mg kg^−1^ (Codex maximum level for Cd). Risk assessment of Cd in consumption of jasmine rice for Thai population was presented. The values of estimated weekly intake (EWI) of jasmine rice for Thai population were 0.25 μg kg^−1^ BW week^−1^ (male) and 0.30 μg kg^−1^ BW week^−1^ (female). The values of estimated monthly intake of 1.07 μg kg^−1^ BW month^−1^ (males) and 1.28 μg kg^−1^ BW month^−1^ (females) were less than 25 μg kg^−1^ BW (the provisional tolerable monthly intake, PTMI). This revealed that it is safe for both Thai males and females in jasmine rice consumption in term of Cd. Moreover, the non-carcinogenic risks evaluated through hazard quotient (HQ) simulations were 0.037 for males and 0.044 for females, indicating no harmful effects from Cd exposure via jasmine rice consumption in the Thai population, as the mean and 90th percentile of the simulated HQ were below the HQ threshold of 1.

## Highlights

• Jasmine rice samples were grown in the northeast of Thailand.
• Cd in jasmine rice from 5 provinces was measured by ICP-MS.
• Cd in jasmine rice samples was lower than the Codex maximum level.
• Estimate daily and weekly intake of Cd was lower for male than female population.
• Non-carcinogenic risk caused by consumption of rice was calculated and simulated.

## Graphical Abstract

**Figure:**
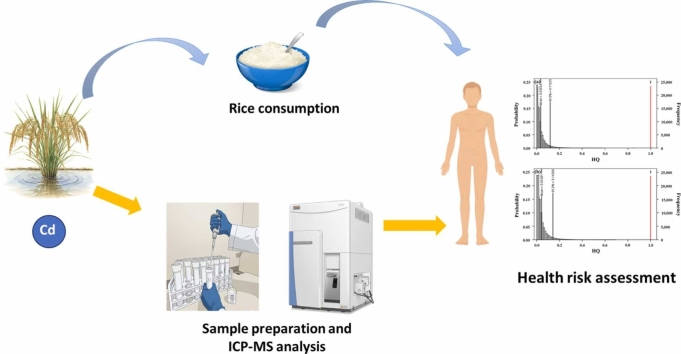

## 1 Introduction

Cadmium (Cd) is a toxic heavy metal that is difficult for the body to eliminate. Because of this, it can accumulate in various tissues, especially the kidneys and liver and lead to the accumulation in other parts of the body. Cd is highly toxic to the kidneys and bones [1], [2], [3], posing significant risks to human health in both short and long term. Cd exposure can enter the body through two routes: the gastrointestinal and the respiratory tracts. Excessive accumulation of Cd leads to renal dysfunction, anemia, hypertension, cardiovascular disorders, memory loss, bone lesions, kidney damage, and abnormalities [4], [5]. Furthermore, Cd is classified as human carcinogen [6].

Sources of the contamination originate from the use of chemicals in agricultural areas. These practices result in heavy metal accumulation, especially Cd contamination in the soil, agricultural products, and the use of water for rice cultivation processes [7]. Cd is contaminated with the use of phosphate fertilizers, herbicide as in the molecular structure of glyphosate which consists of a carboxyl group, an amino group, and a phosphate group with this molecular structure causing glyphosate bind to heavy metals. Long term use or large amounts can lead to the accumulation of heavy metals in the soil, representing a significant source of Cd contamination in agricultural areas [8]. Cd has a long half-life (15–30 years), allowing it to persist in the environment for an extended period, making it highly toxic to plants, animals, and humans. When Cd enters the body, it binds to blood cells and albumin. If the concentration reaches as high as 200 μg, kidney tubular cells are unable to reabsorb low-molecular-weight proteins such as β2-microglobulin. Beside Cd can cause Itai-itai disease, it has also been classified as a human carcinogen [9], [10].

In paddy fields, Cd in the soil exists in the form of Cd^2+^. It moves through the roots to various parts and accumulating in the rice grains that can absorb Cd and eventually accumulates in the rice grain [11]. It has been reported that, although both Cd and As pose a high risk of soil–food chain transfer in paddy rice systems, the ratio of grain-to-soil total elemental concentration for Cd is one to two orders of magnitude higher than that for As. This exceptional mobility poses a significant risk to food safety. Consequently, Cd should be prioritized in food safety monitoring. Maximum level (ML) of Cd in polished rice set by the Codex Alimentarius Commission, Joint FAO/WHO Food Standard Programme is 0.4 mg kg^−1^. The Expert Committee on Food Additives (Joint FAO/WHO Expert Committee on Food Additives - JECFA) has established the safety limit for humans from monthly exposure to Cd (the PTMI) equal to 25 μg kg^−1^ BW [12].

As rice is a staple food in Thailand and a major export product, the country ranks as the world’s second-largest exporter after India [13], Cd contamination in rice is a critical issue that needs to be addressed to maintain both public health and international trade reliability. Jasmine rice, renowned for its premium quality and primarily cultivated in the northern and northeastern regions of Thailand, must be strictly monitored for Cd levels to ensure global consumer confidence.

Risk assessment is related to the uncertainty that might arise from natural variability in an individual's response, variability in the concentration of toxicants, uncertainty in the measurement or estimation of parameters, a lack of precise knowledge. There are two kinds of the models-deterministic and stochastic approaches. In the deterministic model, inputs are represented as point estimates and the uncertainties are ignored. However, the probability distribution of parameters is used as inputs for stochastic approach. For applying stochastic approach in risk assessment, Monte Carlo simulation (MCS) is the most widely used [14].

There are several analytical techniques focusing on the determination of heavy metals in rice such as instrumental neutron activation analysis (INAA) [15], atomic absorption spectrometer (AAS) [16], [17], inductively coupled plasma optical emission spectrometer (ICP-OES) [18], microwave induced plasma atomic emission spectrometer (MP-AES) [19], inductively coupled plasma mass spectrometer (ICP-MS) [14], [17], [20], [21], [22], [23], [24], [25]. Although ICP-MS is the most sensitive technique, sample preparations are significant for heavy metals’ accuracy detection. Traditional sample preparation method used for ICP-MS is acid digestion [17], [18], [19] that is time consuming and is required dangerous reagents. To overcome these, microwave-assisted acid digestion [14], [20], [21], [22], [23], [24], [25] is more accurate and practical method. Therefore, the objective of this study is to evaluate Cd contamination in rice from major Thai agricultural regions, ensuring the safety of domestic consumption and international exports. By utilizing ICP-MS for reliable analysis of Cd in jasmine rice, estimated daily intake (EDI), estimated weekly intake (EWI), and estimated monthly intake (EMI) were subsequently evaluated. Moreover, the potential non-carcinogenic health risks for Thai population were assessed using MCS.

## 2 Materials and methods

### 2.1 Reagents and materials

Ultrapure nitric acid (HNO_3_) and hydrogen peroxide (H_2_O_2_) were obtained from Merck (Darmstadt, Germany). Cd and indium (In) standards were purchased from Agilent Technologies (Santa Monica, CA, USA). In used as an internal standard was added into all working standard solution at the final concentration of 5 μg L^−1^ for the determination of Cd in rice by ICP-MS. Serial dilutions of Cd working standard solution were prepared in 2% HNO_3_. A certified reference material (CRM), SRM 1568b (rice flour) purchased from NIST (Gaithersburg, MD, USA) was used to evaluate the recovery.

### 2.2 Sampling and preparation of rice samples

Forty-six jasmine rice samples were cultivated from Roi Et, Mahasarakham, Surin, Sisaket, and Yasothon provinces in Thailand. Paddy rice samples were cultivated from various farmers’ paddy fields. The rice samples were then sun-dried. The polished rice samples were ground by agate mortar. The powdered rice samples were passed through 250 μm sieve. The powdered rice samples were finally dried for 48 h at 60 °C in an oven and kept in a desiccator. Then, the samples were digested by microwave digestion system and determined using ICP-MS. For method validation, SRM 1568b was also determined by this method.

### 2.3 Microwave digestion and ICP-MS

The powdered rice samples were digested using microwave digestion adapted from the previous method [20]. Shortly, the samples (0.8 g each) were weighed into TFM vessels. The samples were added with 9 mL of ultrapure HNO_3_ and 1 mL of 30% (v/v) H_2_O_2_. After pre-digestion, the closed vessels were digested by CEM MARS 6 microwave digestion system. The digestion program was set at 160 °C for 30 min. The obtained solution was gradually heated to dryness and dissolved in 10.00 mL of 2% (v/v) HNO_3_. The final solution was then filtered through syringe filters (0.45 μm). An appropriate dilution was performed in 2% (v/v) HNO_3_ prior to determination by ICP-MS. The CRM was digested by the same method. The analysis was measured against internal standard, ^115^In. Cd concentrations in the samples were determined in triplicate by Thermo Scientific™ iCAP TQ triple quadrupole ICP-MS (Thermo Fisher Scientific, Waltham, Ma, USA). The conditions for ICP-MS are shown in Table 1. The ICP-MS was operated at an RF power at 1550 W with nickel sample and skimmer cones, plasma gas at 14 L min^−1^, and auxiliary gas at 0.8 L min^−1^. To ensure the accuracy of the analytical method, the method validation followed the Eurachem Guide [26]. The determination of Cd in rice by ICP-MS was validated using rice flour (NIST SRM 1568b) in the same manner as the samples. The measured values were in good agreement with the certified value (0.0224 mg kg^−1^) with the recovery of 85.5%. LOD and LOQ were 0.0058 and 0.013 μg kg^−1^, respectively. Precision of the method in terms of RSD was below 10%.

**Table 1:** Operating conditions for ICP-MS.

**Operating parameters**	**Condition**
RF power (W)	1550
Cool gas flow (L min^−1^)	14
Auxiliary gas (L min^−1^)	0.8
Nebulizer	MicroMist
Spray chamber	Quartz cyclonic spray chamber
Replicates	3
Sample cone and skimmer cone	Ni

### 2.4 EDI, EWI, and EMI

Cd exposure and risk assessment via rice consumption followed the methods described previously. The EDI, EWI, and EMI were calculated by (1), (2), (3), respectively. The EWI and EMI were compared with the PTWI and PTMI, respectively, as established by WHO/FAO expert committee.

(1) $$\mathrm{EDI}=\frac{\left(C\times \mathrm{IR}\right)}{\mathrm{BW}}$$

(2) $$\mathrm{EWI}=\frac{\left(C\times \mathrm{IR}\times 7\right)}{\mathrm{BW}}$$

(3) $$\mathrm{EMI}=\frac{\left(C\times \mathrm{IR}\times 30\right)}{\mathrm{BW}}$$

Where C, IR, and BW represent concentration of Cd (mg kg^−1^), intake rate (g day^−1^), and body weight (kg), respectively. The average intake rate for rice consumption per capita was 205.48 g day^−1^ [27]. The average body weights (BW) for Thai males and females were 68.83 and 57.40 kg, respectively.

### 2.5 Non-carcinogenic risks

Non-carcinogenic risks were determined according to the previously reported method [28]. The risk of Cd from jasmine rice consumption was expressed as the hazard quotient (HQ), calculated using Eq. (4) [16].

(4) $$\mathrm{HQ}=\frac{\mathrm{EDI}}{\mathrm{RfD}}$$

where RfD (mg kg^−1^ day^−1^) is the reference dose of Cd (0.001 mg kg^−1^ BW day^−1^) [17], [21]. MCS, Stochastic approach was applied for risk assessment in this study.

### 2.6 Statistical analysis

MCS was performed by IBM SPSS statistics 23 software. The probabilistic distribution of the HQ was simulated through 100,000 iterations to evaluate non-carcinogenic risk for male and female population in Thailand exposed to Cd via jasmine rice consumption.

## 3 Results and discussion

### 3.1 Cd concentrations and exposure to Cd from jasmine rice consumption

**Fig. 1:**
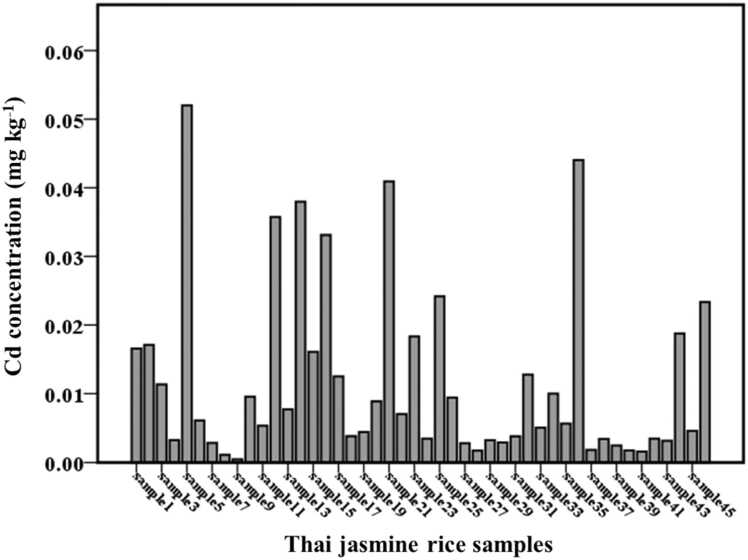
Cd concentration (mg kg^−1^) found in 46 jasmine rice samples.

The Cd concentrations obtained in jasmine rice were 0.00047–0.0520 mg kg^−1^. The mean of Cd concentration obtained in jasmine rice was 0.0119 mg kg^−1^. The distribution of Cd concentration found in jasmine rice is depicted in Fig. 1. Codex ML of 0.4 mg kg^−1^ for Cd in rice was reported [12]. It was found that Cd concentrations in 46 jasmine rice samples were below the ML. The levels of Cd in rice consumed in various countries followed this order: this study (0.0119 mg kg^−1^) < Brazil (0.012 mg kg^−1^) [22] < Iran (0.02 mg kg^−1^) [18] < Indonesia (0.041 mg kg^−1^) [29] < China (0.057–0.11 mg kg^−1^) [30], [31] < Bangladesh (0.18 mg kg^−1^) [23]. The EWI and EMI for jasmine rice consumption were estimated for female and male population in Thailand. The EDI, EWI, EMI, PTWI, and PTMI are presented in Table 2.

**Table 2:** Estimated daily intake (EDI), estimated weekly intake (EWI), estimated monthly intake (EMI), provisional tolerable weekly intake (PTWI), and provisional tolerable monthly intake (PTMI) of Cd exposure by jasmine rice consumption for male and female population in Thailand.

**Thai population**	**EDI (µg kg**^**−1**^**BW day**^**−1**^**)**	**EWI (µg kg**^**−1**^**BW week**^**−1**^**)**	**EMI (µg kg**^**−1**^**BW month**^**−1**^**)**	**PTWI (µg kg**^**−1**^**BW)**	**PTMI (µg kg**^**−1**^**BW)**
Male	0.036 ± 0.039	0.25 ± 0.27	1.07 ± 1.16	7^a^	25
Female	0.043 ± 0.046	0.30 ± 0.32	1.28 ± 1.39		

a The PTWI was withdrawn. The PTMI of 25 μg kg^−1^ BW was established [12].

The EDI values of Cd in jasmine rice consumption for males and females were 0.036 and 0.043 μg kg^−1^ BW day^−1^, respectively. The EWI of Cd was lower for males than for females because of the higher BW of males. The EWI values of males and females for jasmine rice consumption were 0.25 and 0.30 μg kg^−1^ BW week^−1^, respectively, which were roughly 25 times lower than the PTWI. As the PTWI was withdrawn, the PTMI of 25 μg kg^−1^ BW was established by the Codex committee. To compare with the PTMI, the values of EMI were also estimated as summarized in Table 2. The EMI values for females and males were 1.28 and 1.07 μg kg^−1^ BW month^−1^, respectively, both of which were lower than the established PTMI. As a result, jasmine rice consumption in Thailand was considered safe for both males and females in terms of Cd exposure.

### 3.2 Non-carcinogenic risks

**Fig. 2:**
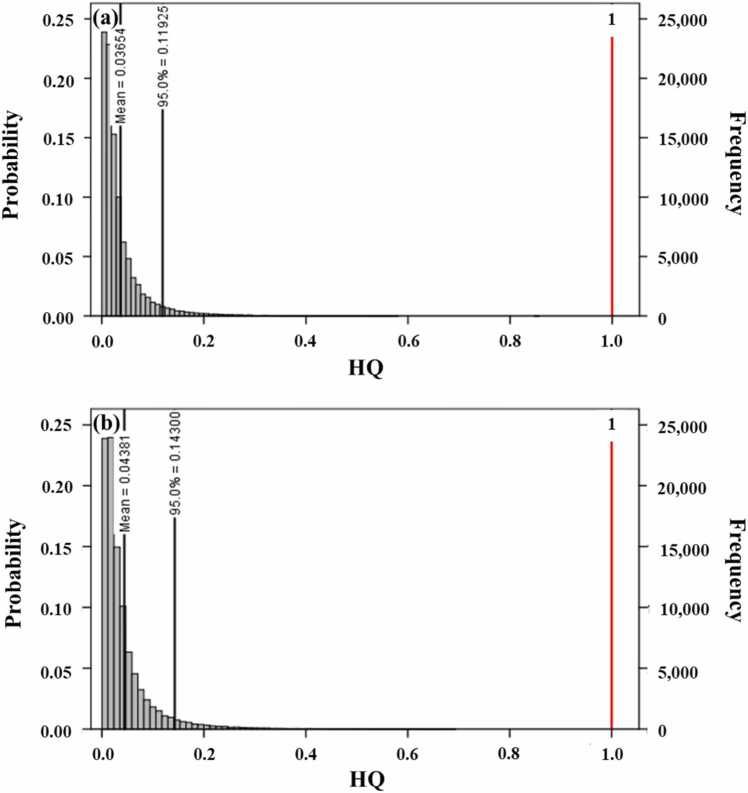
Simulated hazard quotient (HQ) distributions of Cd for non-carcinogenic risks from jasmine rice consumption in Thai (a) male and (b) female population.

The HQ was employed to assess non-carcinogenic risks from Cd exposure based on its probabilistic distribution. Fig. 2 shows the simulated distribution of HQ for Cd. The calculated HQ results including mean, SD, and median are summarized in Table 3. Regarding Cd exposure via jasmine rice consumption, the mean HQ ± SD was 0.037 ± 0.054 and 0.044 ± 0.064 for male and female, respectively. The HQ at 95th percentile of Cd in consumption of jasmine rice for male (Fig. 2a) and female (Fig. 2b) was 0.119 and 0.143, correspondingly. In a comparison of the non-carcinogenic risks from rice consumption across several countries, the order of HQ was India (0.031) [24] < Thailand (this study, 0.037–0.044) < Bangladesh (0.14–0.42) [23], [25] < China (0.29–2.29, except industrial areas) [32].

**Table 3:** Non-carcinogenic risks of Cd through jasmine rice consumption by probabilistic estimation of hazard quotient (HQ) for Thai population.

Thai population	Mean	SD	Median	90th Percentile
Male	0.037	0.054	0.021	0.081
Female	0.044	0.064	0.025	0.097

The mean value of the simulated HQ of Cd for females was higher than that for male population. However, the HQ at 90th percentile for Cd in jasmine rice consumption for male and female of Thai population was less than 1 (the HQ threshold). As a result, there was not associated with harmful effects caused by exposure of Cd via jasmine rice consumption in the Thai population.

## 4 Conclusion

Rice is classified as a primary staple food for more than half of the world's population; therefore, the quality control is essential. In this research, Cd contents in jasmine rice samples consumed in Thailand were measured using ICP-MS. The Cd concentrations in the rice samples were far below the Codex ML. This indicates the good quality of Thai jasmine rice in terms of Cd content. The EWI and EMI values of both males and females were lower than the PTWI and PTMI, respectively. The analysis of health risk of investigated Cd in jasmine rice showed there was no significant non-carcinogenic risk as HQ was far below the HQ threshold (<1) for both Thai males and females. Finally, it is essential to investigate additional toxic elements to ensure food safety.

## CRediT authorship contribution statement

**Supalak Kongsri:** Writing – review & editing, Writing – original draft, Validation, Methodology, Investigation, Formal analysis, Data curation, Conceptualization. **Chunyapuk Kukusamude:** Writing – review & editing, Visualization, Validation, Supervision, Software, Project administration, Methodology.

## Declaration of Competing Interest

The authors declare that they have no known competing financial interests or personal relationships that could have appeared to influence the work reported in this paper.

## Data Availability

The authors do not have permission to share data.

## References

[1] JECFA (Joint FAO/WHO Expert Committee on Food Additives). Evaluation of certain food additives and contaminants: seventy-third report of the Joint FAO/WHO Expert Committee on Food Additives, 2010. Available from: 〈https://apps.who.int/iris/bitstream/handle/10665/44515/WHO_TRS_960_eng.pdf;jsessionid=89F261415D685C32555ABBC949AADA67?sequence=1〉, accessed on Oct 20, 2020.

[2] Rafati Rahimzadeh M., Rafati Rahimzadeh M., Kazemi S., Moghadamnia A.A. (2017). Cadmium toxicity and treatment: an update. J. Intern Med.

[3] WHO (2019).

[4] Singh B.R., McLaughlin M.J. 1999. Cadmium in Soils and Plants. Chapter 10. pp. 257-267. In Launghlin M.J. and B.R. Singh, eds. Cadmium Soils and Plants. Kluwer Academic, Dordrecht.

[5] Hadian Z., Shariatifar N., Arabameri M., Moazzen M., Khaneghah A.M. (2025). Deterministic and probabilistic risk assessment of elemental composition in pistachios and hazelnuts from Iran. Biol. Trace Elem. Res..

[6] Ikeda M., Watanabe T., Nakatsuka H., Moriguchi J., Sakuraji S., Ohashi F. (2015). Cadmium exposure in general populations in Japan: a review. Food Saf..

[7] Han X.Q., Xiao X.Y., Guo Z.H., Xie Y.H., Zhu H.W., Peng C. (2018). Release of cadmium in contaminated paddy soil amended with NPK fertilizer and lime under water management. Ecotoxicol. Environ. Saf..

[8] Jayasumana C., Gunatilake S., Senanayake P. (2014). Glyphosate hard water and nephrotoxic metals: are they the culprits behind the epidemic of chronic kidney disease of unknown etiology in Sri Lanka. Int. J. Environ. Res. Public Health.

[9] Baba H., Tsuneyama K., Yazaki M., Nagata K., Minamisaka T., Tsuda T. (2013). The liver in itai-itai disease (chronic cadmium poisoning): pathological features and metallothionein expression. Mod. Pathol..

[10] Balali-Mood M., Naseri K., Tahergorabi Z., Khazdair M.R., Sadeghi M. (2021). Toxic mechanisms of five heavy metals: mercury, lead, chromium, cadmium, and arsenic. Front. Pharm..

[11] Zhang Q., Zhang L., Liu T., Liu B., Huang D., Zhu Q. (2018). The influence of liming on cadmium accumulation in rice grains via iron-reducing bacteria. Sci. Total Environ..

[12] Codex Alimentarius Commission, Joint FAO/WHO Food Standards Programme, Codex Committee on Contaminants in Foods. CF/14 INF/1. [Internet]. 2021 [cited 2024 Feb 23]. Available from: 〈https://www.fao.org/fao-who-codexalimentarius/sh-proxy/en/%3Flnk%3D1%26url%3Dhttps%25253A%25252F%25252Fworkspace.fao.org%25252Fsites%25252Fcodex%25252FMeetings%25252FCX-735-14%25252FINFO-DOC%25252FCF14_INF01x.pdf&usg=AOvVaw0FfRmiBf1hFLCT8QidjWJP〉.

[13] Principal rice exporting countries worldwide in 2023/2024. [Internet]. 2024 [cited 2024 Feb 23]. Available from: 〈https://www.statista.com/statistics/255947/top-rice-exporting-countries-worldwide-2011/〉.

[14] Djahed B., Taghavib M., Farzadkia M., Norzaee S., Miri M. (2018). Stochastic exposure and health risk assessment of rice contamination to the heavy metals in the market of Iranshahr, Iran. Food Chem. Toxicol..

[15] Kongsri S., Kukusamude C. (2025). Arsenic concentrations in brown rice and health risk assessment. Asia Pac. J. Sci. Tech..

[16] Hensawang S., Chanpiwat P. (2018). Analysis and probabilistic risk assessment of bioaccessible arsenic in polished and husked jasmine rice sold in Bangkok. Chemosphere.

[17] Song D., Zhuang D., Jiang D., Fu J., Wang Q. (2015). Integrated health risk assessment of heavy metals in Suxian County, South China. Int. J. Environ. Res. Public Health.

[18] Ramezani A.M., Hassanabadi M., Naimabadi A., Javan S. (2024). The human health risks assessment posed by the presence of heavy metals in the rice varieties available in the Neyshabur market. Environ. Monit. Assess..

[19] Ozbek N., Tinas H., Atespare E. (2019). A procedure for the determination of trace metals in rice varieties using microwave induced plasma atomic emission spectrometry. Microchem. J..

[20] Kukusamude C., Kongsri S. (2019). Method validation for determination of some rare-earth elements in rice using ICP-MS. J. Phys. Conf. Ser..

[21] Kukusamude C., Sricharoen P., Limchoowong N., Kongsri S. (2021). Heavy metals and probabilistic risk assessment via rice consumption in Thailand. Food Chem..

[22] Barbosa R.M., de Paula E.S., Paulelli A.C., Moore A.F., Souza J.M.O., Batista B.L., Campiglia A.D., Barbosa F. (2016). Recognition of organic rice samples based on trace elements and support vector machines. J. Food Compos. Anal..

[23] Shaheen N., Hasan T., Sultana M., Akhter K.T., Khan I.N., Irfan N.Md, Ahmed Md.K. (2024). Carcinogenic and non-carcinogenic health hazards of potentially toxic elements in commonly consumed rice cultivars in Dhaka city, Bangladesh. PLoS ONE.

[24] Giri S., Singh A.K. (2017). Human health risk assessment due to dietary intake of heavy metals through rice in the mining areas of Singhbhum Copper Belt, India. Environ. Sci. Pollut. Res.

[25] Proshad R., Kormoker T., Islam Md.S., Chandra K. (2020). Potential health risk of heavy metals via consumption of rice and vegetables grown in the industrial areas of Bangladesh. Hum. Ecol. Risk Assess..

[26] Magnusson, B., Ornemark, U., Eds., 2014. Eurachem Guide: The Fitness for Purpose of Analytical Methods – A Laboratory Guide to Method Validation and Related Topics, EURACHEM, 2nd Ed.; pp 1–70.

[27] (2023). Grain and Feed Annual.

[28] U.S. Environmental Protection Agency (U.S. EPA), 1989. Risk Assessment Guidance for Superfund Volume I Human Health Evaluation Manual (Part A) Interim Final (EPA/540/1-89/002). Available from: 〈https://www.epa.gov/sites/production/files/2015-09/documents/rags_a.pdf〉.

[29] Yuliani F., Faridah D.N., Andarwulan N., Srednicka-Tober D. (2025). The Concentration of Potentially Toxic Elements (PTEs) in Indonesian Rice and Their Health Risk Assessment Indonesian Rice and Their Health Risk Assessment. Foods.

[30] Sun Y., Liu S., Liu G., Lu L., Xia Yong (2025). Unregulated heavy metals in Chinese rice pose greater health risks and economic costs than regulated metals. Environ. Sci. Eur..

[31] Jiang Y., Guo H., Chen K., Fei X., Li M., Ma J., He W. (2024). Health risk assessment for potential toxic elements in the soil and rice of typical paddy fields in Henan Province. Toxics.

[32] Ngo H.T.T., Hang N.T.T., Nguyen X.C., Nguyen N.T.M., Truong H.B., Liu C., La D.D., Kim S.S., Nguyen D.D. (2024). Toxic metals in rice among Asian countries: a review of occurrence and potential human health risks. Food Chem..

